# Dr. Richmond B. McKinney

**Published:** 1893-07

**Authors:** 


DR. RICHMOND B. MCKINNEY, of Memphis, was a welcome visitor to the JOURNAL's sanctum last month; Dr. McKinney has charge of the business end of that sterling publication—the *Memphis Medical Monthly*, and having received his medical training and his journal training at the hands of that rare man and extraordinary editor and physician, Prof. Sim, is a *good one*, a "chip of the old block." We predict for this promising young physician and journalist a brilliant career, and wish him all success.

